# Carbon catabolite repression in *Thermoanaerobacterium saccharolyticum*

**DOI:** 10.1186/1754-6834-5-85

**Published:** 2012-11-26

**Authors:** Vasiliki Tsakraklides, A Joe Shaw, Bethany B Miller, David A Hogsett, Christopher D Herring

**Affiliations:** 1 Mascoma Corporation, 67 Etna Road, Suite 300, New Hampshire, 03766, Lebanon

**Keywords:** Catabolite repression, Arabinose, Glucose, HPr, Deoxyglucose, Lignocellulose, Ethanol, Thermophile, Anaerobe

## Abstract

**Background:**

The thermophilic anaerobe *Thermoanaerobacterium saccharolyticum* is capable of directly fermenting xylan and the biomass-derived sugars glucose, cellobiose, xylose, mannose, galactose and arabinose. It has been metabolically engineered and developed as a biocatalyst for the production of ethanol.

**Results:**

We report the initial characterization of the carbon catabolite repression system in this organism. We find that sugar metabolism in *T. saccharolyticum* is regulated by histidine-containing protein HPr. We describe a mutation in HPr, His15Asp, that leads to derepression of less-favored carbon source utilization.

**Conclusion:**

Co-utilization of sugars can be achieved by mutation of HPr in *T. saccharolyticum*. Further manipulation of CCR in this organism will be instrumental in achieving complete and rapid conversion of all available sugars to ethanol.

## Background

Metabolic yield is one of the most important factors in determining economic feasibility for biological-based conversion of biomass to fuels and chemicals. Most bacteria have evolved tailored carbon utilization pathways and regulatory schemes for the uptake and catabolism of carbon sources in their environment. The order in which sugars are utilized is frequently determined by a mechanism known as carbon catabolite repression (CCR) [1]. CCR ensures that the cell’s energy expenditure on sugar import and metabolism will be directed to the carbon source that is most easily accessible and allows for fastest growth [2,3].

The firmicutes are low G+C, gram-positive bacteria and include potential biofuel-producing species from the classes Clostridia and Bacilli. Studies of CCR in firmicutes have revealed the importance of the Histidine-containing Protein HPr [3-5]. HPr(His15-P) donates a phosphate to glucose imported via the phosphotransferase system (PTS). Histidine-dephosphorylated HPr is then phosphorylated by HPr kinase (HPrK) at Ser46 [6-8] and this form of the protein mediates repression in concert with the transcriptional regulator Catabolite Control Protein A (CcpA) [9]. In most firmicutes, His15 and Ser46 phosphorylation of HPr are mutually antagonistic [10,11]. Under conditions of nutrient limitation, HPrK acts as a phosphorylase, removing the serine phosphate of HPr and inhibiting CcpA-mediated gene regulation [8]. A doubly phosphorylated form of HPr has been detected in *Bacillus subtilis* under certain growth conditions; this form of the protein was absent or significantly reduced when strong CCR was induced [12]. Some firmicutes additionally produce Crh (catabolite repression HPr), a protein homologous to HPr but lacking the His15 residue. Crh is involved in CcpA-dependent CCR but plays no role in PTS function [13]. In *Escherichia coli* and other gram-negative enteric bacteria reviewed in [3,14], transcriptional regulation of catabolic genes is mediated by the phosphorylation state of the PTS EIIA^Glc^ subunit and cAMP concentration, not HPr [15,16].

We studied CCR in the firmicute *Thermoanaerobacterium saccharolyticum*, which consumes xylan and other biomass-derived sugars to produce ethanol and a mixture of organic acids [17]. Metabolic engineering has redirected carbon flux almost exclusively to ethanol [18]. In order to develop this organism into an even more efficient biocatalyst, we sought to understand and effectively manipulate its carbon source utilization. *T. saccharolyticum* can grow on a wide spectrum of sugars, and although it can co-utilize glucose and xylose, it exhibits carbon source preferences when cultured in other sugar mixtures. The molecular basis for these preferences is unknown. A study of the glycobiome of the related *Thermoanaerobacter* sp. X514 revealed that glucose and xylose utilization can occur in parallel and are both regulated by transcriptional antiterminators of the BglG family. Hexose and pentose co-utilization was interpreted as absence of CCR in that organism [19]. In the present study, we demonstrate the presence of CCR in *T. saccharolyticum*. We identify homologs to all major CCR genes and find that in *T. saccharolyticum*, CCR is mediated by HPr phosphorylation. We characterize a mutant of HPr that provides relief from CCR and we discuss the possible mechanism by which the mutation leads to the observed growth characteristics.

## Results

### cAMP-independent CCR in *T. saccharolyticum*

**Figure 1:**
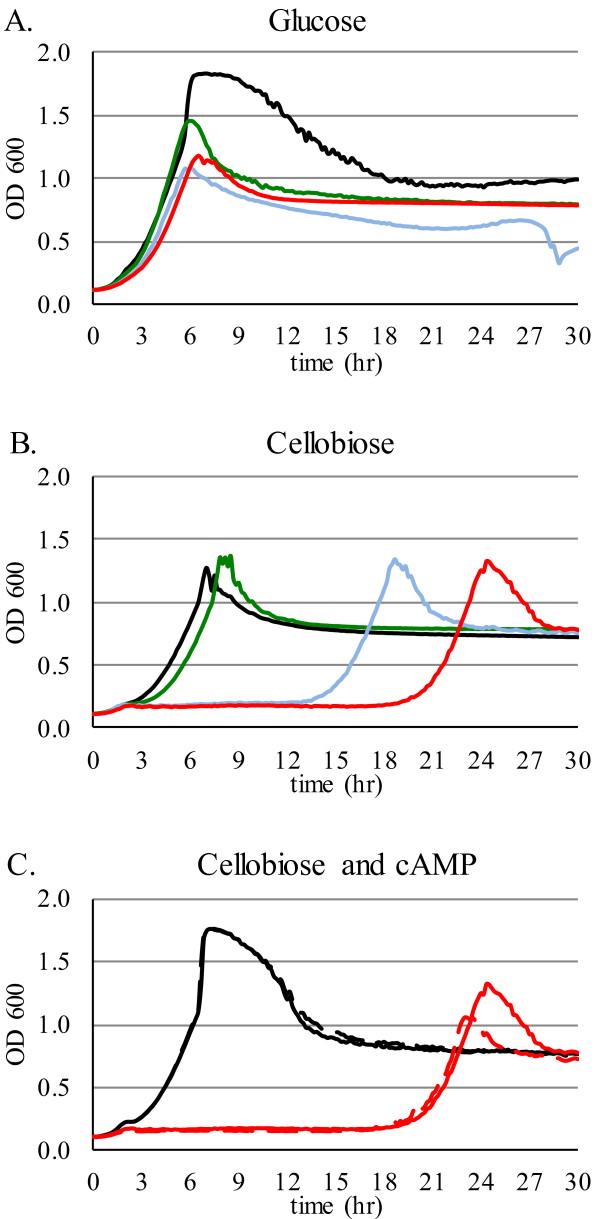
**cAMP**-**independent CCR by 2**-**deoxyglucose.** (**A**) and (**B**): Increasing amounts of 2-deoxyglucose were added to *T. saccharolyticum* M2476 cultures supplemented with 5 g/L glucose or cellobiose (black = 5 mg/L; green = 50 mg/L; blue = 0.5 g/L; red = 5 g/L 2-deoxyglucose). (**C**): cAMP and 2-deoxyglucose were added to *T. saccharolyticum* M2476 cultures supplemented with 5 g/L cellobiose (solid black = no additions; dashed black = 5 mM cAMP; solid red = 5 g/L 2-deoxyglucose; dashed red = 5 mM cAMP and 5 g/L 2-deoxyglucose).

M2476, the parent strain used in this study, was derived from the ethanologen strain M1442 [20] by deletion of the *perR* gene. Deletion of *perR* upregulates the oxidative stress response and improves oxygen tolerance [21] but is not expected to influence CCR. The non-metabolizable glucose analog 2-deoxyglucose has been used extensively to induce catabolite repression in bacteria [22-27]. When utilization of the sole carbon source in the medium is repressed, growth is inhibited and CCR can be detected by monitoring the culture optical density [23]. We added 2-deoxyglucose to microwell cultures containing a single sugar as the main carbon source. 2-deoxyglucose delayed growth in cellobiose (Figure 1B) but not glucose cultures (Figure 1A), indicating that 2-deoxyglucose led to CCR of cellobiose utilization. Exogenous addition of cAMP provides CCR relief in *E. coli*[28,29]. 5 mM cAMP had no effect on the growth delay caused by 2-deoxyglucose (Figure 1C) suggesting that in *T. saccharolyticum*, 2-deoxyglucose-induced CCR is cAMP-independent.

### *T. saccharolyticum* CCR is mediated by HPr

**Figure 2:**
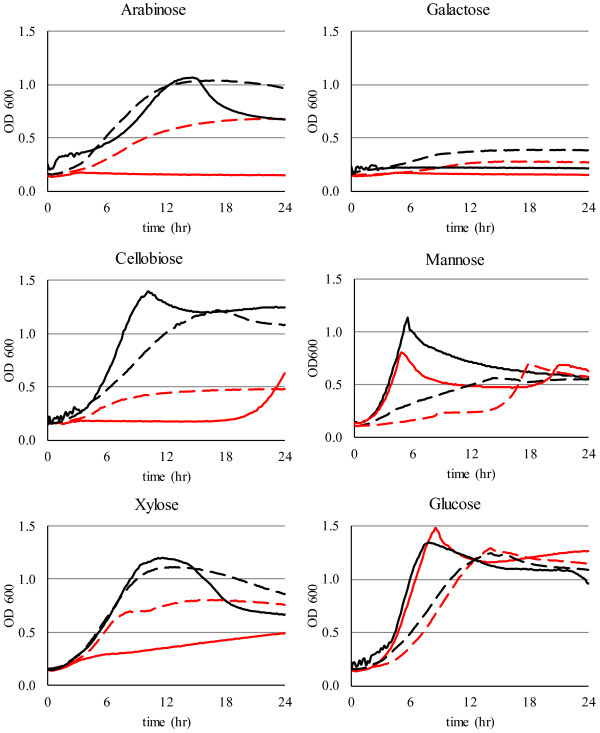
***T. saccharolyticum *CCR is relieved by HPr His15Asp.** Cultures of strains M2476 (HPr, solid lines) and M2907 (HPr His15Asp, dashed lines) supplemented with the indicated sugars were grown in the absence (black lines) or presence (red lines) of 5 g/L 2-deoxyglucose.

A BLAST search of the *T. saccharolyticum* genome with *B. subtilis* CCR genes showed significant matches for HPr (E-value: 3 × 10^-17^), HPrK (1 × 10^-85^), Crh (9 × 10^-24^) and CcpA (16 matches with E-values: 3x10^-19^-1 × 10^-85^) suggesting that *T. saccharolyticum* uses the same elements of CCR as *B. subtilis*. To study the role of HPr in *T. saccharolyticum* CCR, we replaced the endogenous HPr gene in strain M2476 with a modified copy encoding the histidine phosphomimetic HPr His15Asp to create strain M2907. We reasoned that if HPr mediates PTS import and CCR, the mutant strain would be compromised for PTS sugar import and exhibit resistance to CCR. We evaluated growth of strains M2476 and M2907 on glucose, xylose, mannose, galactose, arabinose or cellobiose as the primary carbon source. Growth of the mutant strain M2907 in all sugars tested (Figure 2, dashed black lines) indicates that these sugars can be imported independently of HPr as a PTS phosphate donor. M2907 grew more slowly than M2476 on glucose, mannose and cellobiose, likely due to reduced PTS function in the mutant strain. M2907 grew faster than its parent on xylose, galactose, arabinose and cellobiose in the presence of 2-deoxyglucose (Figure 2, red lines) suggesting that CCR of these sugars is relieved by the HPr His15Asp mutation. While other ethanologen strains of *T. saccharolyticum* grow well on galactose, M2476 does not, which was confirmed in bottle cultures. The HPr His15Asp mutation slightly improved growth in galactose.

### HPr His15Asp leads to altered order of sugar utilization

**Figure 3:**
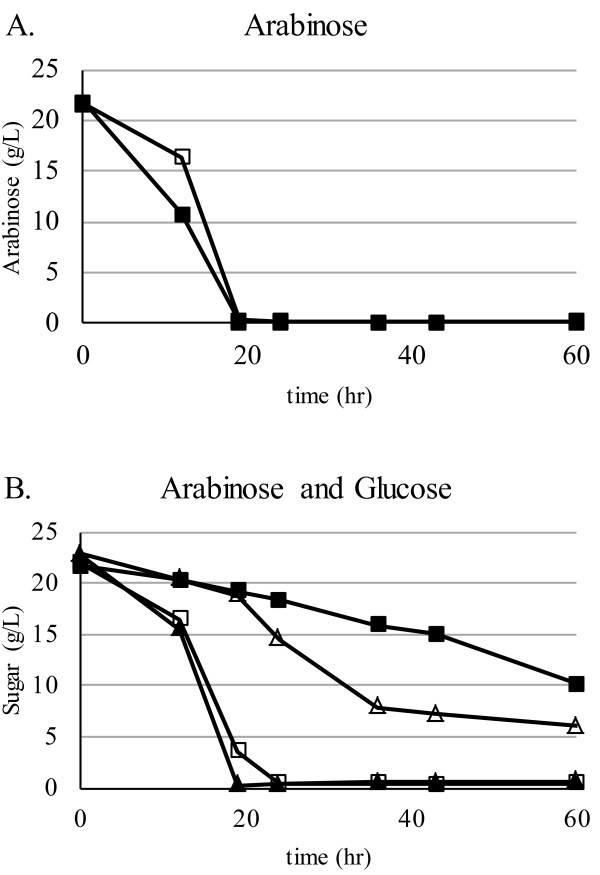
**HPr His15Asp mutation enables arabinose utilization in the presence of glucose.** The level of glucose (triangles) and arabinose (squares) remaining in the culture medium was determined for cultures of *T. saccharolyticum* strains M2476 (HPr, filled symbols) and M2907 (HPr His15Asp, open symbols) supplemented with (**A**) arabinose or (**B**) arabinose and glucose.

We next examined the effect of the HPr His15Asp mutation on sugar utilization in a mixture of sugars. Experiments were carried out in anaerobic bottles (data not shown) and fermentors (Figure 3) with similar results. When arabinose was the primary carbon source, M2476 and M2907 consumed arabinose at a similar rate and depleted it from the medium by 20 hours (Figure 3A). In a mixture of glucose and arabinose, M2476 preferentially consumed glucose within the first 20 hours and had only consumed 50% of the arabinose by 60 hours (Figure 3B, filled symbols). The M2907 mutant showed complete reversal of sugar preference: arabinose was utilized at the same rate as when it was supplied alone and glucose was only partially utilized by 60 hours (Figure 3B, open symbols). A strain carrying mutations in HPr at positions 45 and 46 was unable to grow on arabinose (Additional file 1).

**Figure 4:**
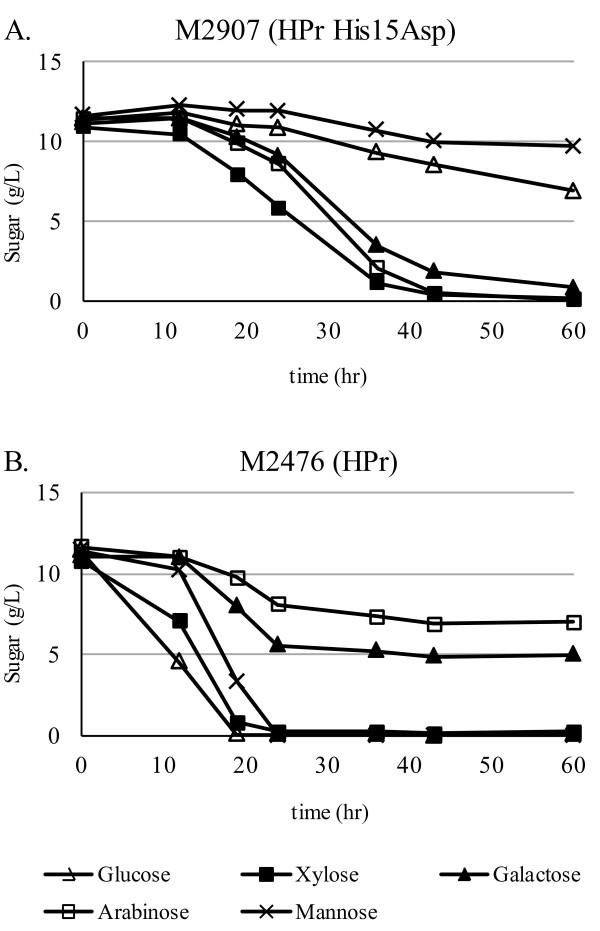
**HPr His15Asp mutation alters the order of sugar utilization.** The level of sugar remaining in the culture medium was determined for cultures of *T. saccharolyticum* strains (**A**) M2476 and (**B**) M2907 supplemented with glucose, arabinose, xylose, galactose and mannose, as indicated.

When the same strains were grown in a mixture of glucose, arabinose, galactose, mannose and xylose, M2907 again demonstrated an altered sugar utilization profile (Figure 4). Galactose and arabinose consumption was clearly derepressed and was complete by 60 hours. Glucose utilization was significantly slower in the mutant (Figure 4A) in agreement with the previous experiment (Figure 3B). Mannose and xylose utilization presented a more complex phenotype. Both these sugars were consumed in the presence of glucose by the wild-type strain (Figure 4B). Consumption of mannose was largely inhibited in strain M2907. In contrast, xylose was consumed to completion at a rate similar to that for arabinose and galactose.

## Discussion

The ability of *T. saccharolyticum* to ferment biomass-derived sugars at elevated temperature renders it an attractive biocatalyst for the production of fuels and renewable chemicals. We investigated carbon catabolite repression in this organism to better understand sugar utilization and how to manipulate it. Sequence homology and our experimental data indicate that *T. saccharolyticum* CCR is regulated by HPr in a mechanism similar to that of the model organism *B. subtilis*. We have identified orthologues to all the major elements in the *B. subtilis* pathway: HPr, HPrK, Crh and CcpA. HPr-mediated CCR is emerging as a shared feature of the firmicute phylum with evidence of its presence in all firmicutes studied to date [3].

In our experiments, the HPr His15Asp mutation led to relief of 2-deoxyglucose-induced repression for multiple sugars and allowed for arabinose and galactose utilization in the presence of glucose. We speculate that HPr His15Asp leads to CCR relief through lowering glucose/2-deoxyglucose import and preventing CcpA activation. Lack of a donor phosphate to transfer from HPr(His-P) to the PTS EIIA^Glc^ subunit is expected to lead to reduced PTS function and therefore limit any downstream repressive effects of glucose import. In *Listeria monocytogenes*, a His15Ala or His15Asp HPr mutation leads to reduction of HPr PTS activity by more than 96% [30]. Consistent with this result, M2907 grew more slowly on glucose (Figure 2) and consumed glucose at a slower rate (Figures 3 and 4). Reduced glucose import may therefore be responsible for CCR relief in the histidine-substituted HPr mutant strain. HPr His15Asp may also provide relief from CCR by directly preventing CcpA activity. The antagonistic relationship of Ser46 and His15 phosphorylation opens the possibility that the histidine phosphomimetic mutant blocks serine phosphorylation of HPr [11] and therefore prevents activation of CcpA by HPr(Ser-P). *In vitro* evidence has shown that *Streptococcus salivarius* and *L. monocytogenes* HPr(His15-P) or its phosphomimetic variant HPr(His15Asp) can be phosphorylated at the serine position [30,31] and a doubly phosphorylated form of HPr has been detected in *B. subtilis*[12]. However, the level of doubly phosphorylated HPr is reduced under CCR [12] and doubly phosphorylated HPr does not bind CcpA [32,33]. This is likely because unphosphorylated His15 contributes to HPr-CcpA binding directly and phosphorylation at His15 blocks CcpA activation by disrupting this interaction [32]. The phosphomimetic mutant HPr His15Asp reported here is therefore expected to disrupt HPr-CcpA interaction and block downstream CcpA-mediated repression. Whether by controlling intracellular concentration of the catabolite repressor or the activity of CcpA, HPr is clearly a major regulator of CCR in *T. saccharolyticum*.

Growth of the mutant strain in all sugars tested suggests that phosphate transfer from HPr(His-P) is not essential in the metabolism of glucose, xylose, mannose, galactose, arabinose and cellobiose. The *T. saccharolyticum* genome annotation predicts that arabinose and xylose are likely transported by ABC-type transporters rather than the PTS. We have identified putative orthologues of GlcU and GlkA, a glucose importer and glucokinase [34-37], as candidates for non-PTS glucose transport.

Although growth on mannose and xylose was repressible by 2-deoxyglucose (Figure 2), both these sugars were consumed by the wild-type *T. saccharolyticum* strain inoculated in medium containing glucose (Figure 4B), suggesting that glucose affects the utilization of these sugars to a lesser extent. Whereas the non-metabolizable 2-deoxyglucose effectively shuts down their metabolism, glucose is a consumable repressor and its intracellular concentration may be lower. Alternatively, cells actively growing on glucose may contain metabolic intermediates that lessen CCR of xylose and mannose. Supporting this, consumption of xylose and mannose quickly followed after an initial reduction of glucose in the medium, followed by significant overlap in utilization of all three sugars. In the HPr His15Asp strain, xylose was completely consumed at a slower rate than in the wild-type strain but at a similar rate to arabinose and cellobiose. Mannose utilization was very poor in the HPr His15Asp strain, suggesting that it is likely transported by the same or similar PTS components as for glucose. The data show three groups of sugars with similar CCR and utilization profiles: 1) glucose and mannose - both dependent on phosphoryl transfer at His15 of HPr; 2) xylose – affected to some degree by CCR but partly independent; and 3) arabinose and galactose – both apparently utilized best when HPr / PTS function is blocked.

HPr has also been implicated in pathway-specific activation or repression. *Lactobacillus brevis* HPr(Ser-P) inactivates lactose and galactose permease [38,39]. Reduced serine phosphorylation due to a phosphomimetic histidine mutation is therefore expected to relieve repression of these sugars. In *Streptococcus thermophilus*, HPr(His-P) is required for activation of lactose transport through activating phosphorylation of the lactose permease [40]. When glucose is present and imported by the PTS, phosphate is diverted to glucose preventing activation of lactose import. As the HPr His15Asp mutant is unable to donate a phosphate, any such sugar import pathways requiring phosphate transfer from HPr(His-P) will remain inactive. Study of HPr interactions with sugar permeases is required to establish whether it plays such sugar-specific roles in *T. saccharolyticum*.

Although derepressed for arabinose and galactose utilization in the presence of glucose, the HPr His15Asp strain was not able to co-utilize all five sugars present in biomass efficiently because of a detrimental effect of the HPr mutation on glucose and mannose utilization. It should be possible to overcome this limitation by selection for spontaneous glucose and mannose utilizing isolates [41]. Alternatively, expression of heterologous high-capacity transporters or manipulation of native transporters may serve to decouple preferred-sugar transport and CCR.

## Conclusions

Preferential utilization of carbon sources offers a selective advantage in nature where bacteria compete for resources [2,3] but hinders industrial applications where metabolic yield and productivity determine economic feasibility. Understanding the components and effectors of CCR is critical to the development of biocatalysts with expanded carbon source utilization profiles [42]. In the present study, we have begun to characterize the molecular basis of CCR in *T. saccharolyticum*. We found that *T. saccharolyticum* CCR is mediated by HPr and used our findings to construct a CCR derepressed strain. Further study and manipulation of *T. saccharolyticum* CCR should enable the engineering of strains that co-utilize mixed sugars to achieve increased metabolic yield and wider feedstock utilization.

## Methods

### Strains and media

M2476, the parent strain used in this study, was derived from the ethanologen strain M1442 [20] by deletion of the *perR* gene (Tsac_2491 in *T. saccharolyticum* genome [GenBank: CP003184]) using previously described methods [43-45]. We replaced the endogenous HPr gene in strain M2476 with a modified copy encoding the HPr His15Asp mutant using a previously described method for genomic integration [44] to create strain M2907. All growth experiments were conducted in TSC7 medium (per liter: 8.5 g yeast extract, 1 g trisodium citrate*2H_2_O, 1 g KH_2_PO_4_, 2 g MgSO_4_*7H_2_O, 1.85 g (NH_4_)_2_SO_4_, 0.2 g CaCl_2_*2H_2_O, 0.2 g FeSO_4_*7H_2_O, 0.12 g Methionine, 0.5 g L-Cysteine HCl, pH 5.8) supplemented with the sugars indicated in the text at 55°C under anaerobic conditions. Although *T. saccharolyticum* can utilize yeast extract as a carbon source, the amount contained in TSC7 is not sufficient to attain the optical densities observed in these assays without the addition of a sugar carbon source.

### 2-deoxyglucose-induced repression assays

For 2-deoxyglucose titration, a range of 0.005 – 5.0 g/L 2-deoxyglucose and 5 mM cAMP (Sigma-Aldrich, St. Louis, MO) were added as indicated to *T. saccharolyticum* microwell cultures supplemented with 5 g/L glucose or cellobiose, inoculated with 10% v/v of an exponentially growing culture in the same medium (one of three replicate experiments is presented in Figure 1). To determine the effect of HPr His15Asp mutation on 2-deoxyglucose-induced CCR, microwell cultures supplemented with 10 g/L glucose, xylose, mannose, galactose, arabinose or cellobiose were inoculated with 10% v/v exponentially growing culture of M2476 or M2907 (one of two replicates is shown in Figure 2). Growth curves were obtained by measuring absorbance at 600 nm in a BioTek PowerWaveXS plate reader with intermittent agitation, inside a COY laboratories anaerobic chamber with a nominal atmosphere of 85% N_2_, 10% CO_2_, 5% H_2_.

### Sugar co-utilization tests

Experiments were carried out in anaerobic bottles (data not shown) and fermentors (Figure 3) with similar results. Sartorius Biostat Aplus fermentors at 1 liter working volume were sparged with 95% N_2_, 5% CO_2_ gas prior to inoculation with 5% v/v overnight culture grown in the same medium. Agitation was at 150 rpm and the pH was maintained at 5.8 with NH_4_OH. Sugars in the culture medium were analyzed by HPLC using a BioRad Aminex HPX-87P column and a refractive index detector. For the two-sugar fermentations, glucose, arabinose or both sugars were added to a final concentration of 20 g/L each in TSC7 medium. For the five-sugar fermentations, glucose, arabinose, galactose, mannose and xylose were added to a final concentration of 10 g/L each in TSC7 medium.

## Abbreviations

Asp: Aspartate; BLAST: Basic local alignment search tool; cAMP: Cyclic adenosine monophosphate; CcpA: Catabolite control protein A; CCR: Carbon catabolite repression; Crh: Catabolite repression HPr; His: Histidine; HPr: Histidine-containing protein; HPrK: HPr kinase; PTS: Phosphotransferase system; Ser: Serine.

## Competing interests

The authors are current or former employees of the Mascoma Corporation, which has a commercial interest in the organisms used in this study.

## Authors' contributions

VT participated in construction of strain M2476, constructed strain M2907, carried out 2-deoxyglucose CCR experiments and drafted the manuscript. AJS and BBM carried out the sugar co-utilization experiments and participated in construction of strain M2476. CDH coordinated the study and helped to draft the manuscript. DAH supervised the execution of the study. All authors read and approved the final manuscript.

## Supplementary Material

Additional file 1 Comparison of two HPr mutations. Click here for file
