# Supplementary material for: Carbon catabolite repression in Thermoanaerobacterium saccharolyticum
Source: Biotechnol Biofuels. 2012 Nov 26;5:85. doi: 10.1186/1754-6834-5-85 (PMC3526391; doi:10.1186/1754-6834-5-85)
Supplement: Additional file 1 — Comparison of two HPr mutations. [file 1754-6834-5-85-S1.docx]

**Supplemental Material: Comparison of two HPr mutations**

We replaced the endogenous HPr gene in strain M2476 with a modified copy encoding HPr with a Ser46Ala substitution using a previously described method for genomic integration and marker removal [44] to create strain M2906. Sequencing of the HPr genomic region in a precursor to M2906 revealed the inadvertent introduction of a mutation resulting in an additional Lys45Arg substitution. This substitution was traced to a mutation introduced during the construction of the plasmid used for mutagenesis. 96-well format microtiter plate cultures in TSC7 medium supplemented with 10 g/L glucose, xylose or arabinose were inoculated with 10% v/v of cultures of M2476, M2906 or M2907 growing exponentially in 10 g/L glucose. Growth curves were obtained by measuring absorbance at 600 nm in a BioTek PowerWaveXS plate reader with intermittent agitation, inside a COY laboratories anaerobic chamber with a nominal atmosphere of 85% N_2_, 10% CO_2_, 5% H_2_ at 55^o^C.

As shown in the data below, growth of M2906 in glucose was delayed, but markedly better than the His15Asp mutant. Very poor growth was seen with M2906 in xylose. No growth was observed in arabinose, even when other sugars such as glucose were present (data not shown). We expected arabinose utilization to be derepressed by the Ser46Ala mutation due to the resulting HPr’s inability to interact with CcpA. Growth inhibition from the presence of arabinose was surprising, but may still be consistent with CCR derepression. The apparent toxicity may be due to accumulation of arabinose intermediates from uncontrolled transcription of the arabinose gene cluster, as was hypothesized for an AraR-null mutant in *Bacillus subtilis* [46]. Taken together, the growth profiles of the two HPr mutants suggest that the His15Asp mutation compromises glucose utilization more severely, but both result in significant growth inhibition in certain conditions. Neither mutation abolished CCR in a completely satisfactory way, likely because of positive and negative pleiotropic effects.

44. Shaw AJ, Covalla SF, Hogsett DA, Herring CD: **Marker removal system for *Thermoanaerobacterium saccharolyticum* and development of a markerless ethanologen.** *Applied and environmental microbiology* 2011, **77:**2534-2536.

46. Inácio JM, Costa C, de Sá-Nogueira I. **Distinct molecular mechanisms involved in carbon catabolite repression of the arabinose regulon in *Bacillus subtilis*.** *Microbiology* 2003, **149:**2345-2355.

Supplementary Figure
